# A Novel Interference Avoidance Based on a Distributed Deep Learning Model for 5G-Enabled IoT

**DOI:** 10.3390/s21196555

**Published:** 2021-09-30

**Authors:** Radwa Ahmed Osman, Sherine Nagy Saleh, Yasmine N. M. Saleh

**Affiliations:** 1 Basic and Applied Science Department, College of Engineering and Technology, Arab Academy for Science and Technology (AAST), Alexandria 1029, Egypt; 2 Computer Engineering Department, College of Engineering and Technology, Arab Academy for Science and Technology (AAST), Alexandria 1029, Egypt; 3 Computer Science Department, College of Computing and Information Technology, Arab Academy for Science and Technology (AAST), Alexandria 1029, Egypt; yasmine_nagi@aast.edu

**Keywords:** IoT, 5G, interference, deep learning, 1D-CNN, throughput, energy efficiency, optimization

## Abstract

The co-existence of fifth-generation (5G) and Internet-of-Things (IoT) has become inevitable in many applications since 5G networks have created steadier connections and operate more reliably, which is extremely important for IoT communication. During transmission, IoT devices (IoTDs) communicate with IoT Gateway (IoTG), whereas in 5G networks, cellular users equipment (CUE) may communicate with any destination (D) whether it is a base station (BS) or other CUE, which is known as device-to-device (D2D) communication. One of the challenges that face 5G and IoT is interference. Interference may exist at BSs, CUE receivers, and IoTGs due to the sharing of the same spectrum. This paper proposes an interference avoidance distributed deep learning model for IoT and device to any destination communication by learning from data generated by the Lagrange optimization technique to predict the optimum IoTD-D, CUE-IoTG, BS-IoTD and IoTG-CUE distances for uplink and downlink data communication, thus achieving higher overall system throughput and energy efficiency. The proposed model was compared to state-of-the-art regression benchmarks, which provided a huge improvement in terms of mean absolute error and root mean squared error. Both analytical and deep learning models reached the optimal throughput and energy efficiency while suppressing interference to any destination and IoTG.

## 1. Introduction

The fifth generation (5G) is considered a basic and emerging technique for the Internet-of-Things (IoT). IoT is a communication environment where a massive number of devices communicate with each other. IoT devices can be included in smart homes, healthcare, and industrial and autonomous vehicles, which improve people’s daily life [1]. 5G networks address the major challenges that exist in cellular networks. They enable all devices to communicate with each other without the need for a base station (BS) which is known as device-to-device (D2D) communication. Furthermore, they enable machine-to-machine (M2M) and device-to-everything (D2E). In addition, 5G authorizes secure, low-latency, reliable and efficient connectivity, also supporting mobility [2]. One of the most important systems that deploy D2D communication nowadays is 5G-enabled IoT which is considered a promising future technique. 5G-enabled IoT communication to supports a large number of applications such as self-driving cars, drones, virtual reality, security surveillance, and many more applications [3]. All the devices used in these applications communicate with each other or with an access point or infrastructure using wireless or wired links [4]. Compared to wired, wireless links are more suitable and efficient to be used for IoT devices. Additionally, wireless links provide a high rate and reliability with low latency. 

5G-enabled IoT is expected to face many challenges such as security, privacy, data control, latency, interference, resource allocation and power consumption. In the literature, diverse solutions have been proposed to overcome those potential problems, yet some challenges still need further investigation [5]. IoT generates an enormous amount of data that have to be processed using machine learning and data analytics [6]. Machine learning, which is a branch of Artificial Intelligence (AI), focusses on how to extract hidden information in the provided data and to help to make decisions. One of the famous machine learning techniques, which has gained a lot of interest lately, is deep learning. For the past decade, deep learning has gained a lot of attention as its tremendous powers have built models that can learn tough problems and achieve high performance. Various recent 5G-enabled IoT applications have adopted deep learning since it can provide higher level of analytical processing, and thus dramatically enhancing results [7,8]. 

IoT devices (IoTDs) and cellular users equipment (CUEs) transmit and receive data while sharing the same spectrum in 5G-enabled IoT. Usually IoTD communicates with an IoT gateway (IoTG): However CUE communicates with base station (BS) or other CUE which is known as D2D communication. All devices send data using the same spectrum, causing interference at BS or any CUE receiver and IoTG. This interference affects the system reliability and efficiency. In this work, an interference avoidance scheme using a deep learning model is proposed. The main goal of the proposed model is to increase the overall system throughput and energy efficiency. The contributions of this article are summarized as follows:

- The proposed approach developed an efficient method to enhance the overall system performance in terms of system throughput and energy efficiency.
- An optimization problem using an analytical and deep learning model was formulated to ascertain the reliability and efficiency of communication among 5G and IoTs.
- The proposed approach aims to decrease or eliminate the interference in 5G networks and IoT systems. This was achieved through determining the optimum distance between CUE-IoTG and IoTD-D for the uplink (UL) data communication and between BS-IoTD and IoTG-CUE for the downlink (DL) data communication. This can be achieved based on different parameters, which affect the system performance such as transmission power, distance between CUE-D and IoTD-IoTG, path loss and signal-to-interference-plus-noise ratio (*SINR_th_*).
- The proposed approach allowed the transmission of CUE and IoTD, using a deep learning model, to predict the suitable acceptable distance between CUE-IoTG and IoTD-D (uplink) and between BS-IoTD and IoTG-CUE (downlink) thus avoiding severe interference.
- The proposed deep learning model was compared to state-of-the-art benchmark methods and it provided a marked improvement in the results.
- The proposed model can be used in the design phase for interference prediction and circumvention.
- The proposed approach was investigated in terms of overall system throughput and energy efficiency under different conditions, such as the path loss exponent, transmission power, different *SINR_th_* values, and different transmission ranges. The whole network can be optimized by these findings in a vibrant environment.

The upcoming sections are organized as follows: First the related work of IoT and 5G networks will be presented in Section 2. The proposed analytical and deep learning models will be thoroughly discussed in Section 3. Section 4 will show the details of all the experimental work and analytics of the results. Finally, the presented work will be summarized and concluded in Section 5. 

## 2. Related Work

Decreasing the interference in IoT communication and 5G is an important issue that should be tackled, as it directly affects the system performance. In the literature, considerable research effort and solutions have been dedicated for the minimization of interference among devices in IoT-based networks such as optimization [9,10,11,12,13,14], deep learning [15,16,17,18,19,20,21,22], modulation [23,24] and the Nash nocoopertaive power game [25].

In [9], a stochastic optimization problem for a network of IoT devices and cellular users sharing the same frequency spectrum with drones was formulated to obtain the optimized transmission power and maximize energy efficiency given lower interference constraints. In addition, the proposed idea in [10] was based on optimizing the random-access procedure, which allowed users to send messages to the base station using one of the target receiver powers according to which the base station could differentiate between different users using successive interference cancellation. Furthermore, the authors of [11] proposed an approach for the resource allocation and placement of multiple unmanned aerial vehicle base stations in an uplink IoT network. Their approach was divided into three main steps: k-means clustering algorithm to group devices served by the same base station, subchannel assignment allocation for devices to diminish interference, and optimization of the transmission power of the IoT devices and the altitudes of the unmanned aerial vehicles. Moreover, an analysis of a hybrid transceiver design problem was presented in [12] for the maximization of the energy efficiency of multiple-input multiple-output interference channels for IoT power constraint devices. The authors of [13] proposed a utilization of Piece-Wise and Forward Non-Orthogonal Multiple Access (PF-NOMA) in a cooperative communication based on an optimization problem. Their proposed model acquired the optimal power and time splitting factors to achieve the maximum rates. Additionally, [14] proposed a new framework called the interference control model. This proposed model aimed to control the interference among IoT and 5G networks based on an optimization technique to maximize the system efficiency and reliability.

The deployment of deep learning for the minimization of interference was presented in [15] where spectral efficiency was enhanced using a scheme based on deep reinforcement learning, which reused the spectrum resources in the communication of the D2D and cellular user equipment (CUE). Similarly, the authors of [16] aimed to enhance spectral efficiency. They proposed the deployment of multiple concurrent frequency bands instead of one channel where deep leaning was used to dynamically select the most suitable channel based on quality requirements such as the signal-to-interference-plus-noise-ration. In addition, in [17], an interference control scheme based on reinforcement learning was proposed to allow the base station to optimize its downlink transmission power while being oblivious of the distribution of the inter-cell interference. The application of Deep Reinforcement Learning (DRL) in which each subcarrier power allocation could be adjusted among D2D pairs was proposed in [18] to reduce latency and increase reliability in D2D communication while rigorous interference constraints were satisfied. Furthermore, the authors in [19] considered deep learning to highlight the interference problem among D2D communications in cellular-enabled IoT networks. The proposed model highlighted the delayed latency and burden on enhanced node base station nodes. Moreover, [20] proposed a scheme to protect energy efficient video transmission in an IoT system against interference using reinforcement learning. In the proposed scheme, a base station managed the IoT transmission action such as transmission power, rate of encoding and scheme of modulation and coding with no knowledge of the transmission channel model. An investigation of different scenarios for channel access management of indoor IoT communication was presented in [21], in which a distributed coordination scheme, based on reinforcement learning, allowed devices to learn to control their activity patterns based on the deep learning. The effect of imperfect inference cancelation and constraint transmission power requirements were studied in [22] where NOMA and packet diversity were jointly adopted. 

Other approaches have been adopted, such as in [23], where a modulation scheme was used for 4G and 5G to obtain the required frequency offset to ensure their coexistence with minimal interference among adjacent channels. In addition, in [24], a multiplexing technique was presented for D2D communication to enhance resource utilization and minimize interference of the D2D users with the cellular users. The proposed technique divided each cell into two regions and spectrum resources were allocated to each region to reduce interference among adjacent cells. Finally, in [25], the authors proposed a novel scheme based on the Nash noncooperative power game for a multiuser multiple-input multiple-output power downlink for handling the interference among IoT devices. 

A lot of research has been proposed for the performance enhancement of 5G-enabled IoT systems, yet there still is room for further investigations focusing on how IoTDs, CUEs, BS and IoTG interact to decrease the interference occurrence at D and IoTG for uplink and CUE and IoTG for downlink. The main goal of this work is to determine the required conditions to decrease or eliminate the interference in 5G networks and IoT systems. This can be achieved by establishing the minimum appropriate predicted distance between CUE-IoTG and IoTD-D for uplink data communication and between BS-IoTD and IoTG-CUE for downlink data communication in order to decrease interference at any D and IoTG. The proposed model deploys deep learning and analytical optimization in which a distributed deep learning model for IoT and 5G networks is used to learn how all of IoT and CUE devices, BS and IoTG can avoid interference by adapting the distance between CUE-IoTG and IoTD-D for uplink and the distance between IoTG-CUE and BS-IoTD for downlink. This enhances the system reliability and efficiency. The assessment of the overall system performance is determined in terms of system throughput and energy efficiency.

## 3. Proposed Model

In this section, the proposed model for controlling the interference affecting each destination is described by a numerical optimization technique. Next, the dataset generation based on the proposed analytical model is demonstrated followed by a proposed deep neural network architecture that would be applied to sending devices, base stations and IoT gateways in real life.

### 3.1. System Model and Problem Formulation

**Figure 1:**
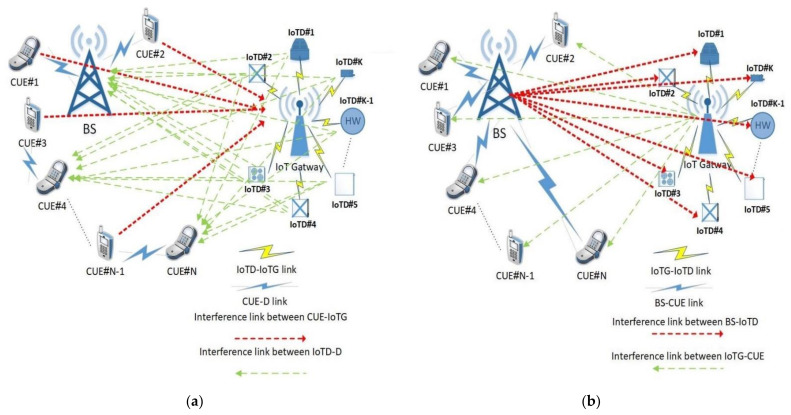
Proposed network schematic. (**a**) Uplink data communication (**b**) Downlink data communication.

The proposed network assumes that there are N number of CUEs, K number of IoTDs, a BS for cellular communication and an IoTG for IoT communication sharing the same spectrum as shown in Figure 1. Figure 1a shows the uplink (UL) data communication for cellular network and IoT communication. There are two means of communication for the cellular network: (i) the CUE communicates with other CUEs, which is known as D2D communication, or (ii) the CUE communicates with the base station, which is the standard known cellular communication. Additionally, there are a number of IoTDs communicating directly with IoTG. Assume that at least one CUE and IoTD sharing the same spectrum have information that needs to be transmitted to the destination makes the BS and any destination node suffer from interference caused by all the transmitted sources. During the downlink (DL) as shown in Figure 1b, BS and IoTG transmit data to CUEs and IoTDs, respectively; in this case, the interference occurs at any CUE and IoTD. The aim of the proposed model is to control the interference among all destinations to enhance the overall network performance by optimizing the system throughput $\left(S\right)$ and energy efficiency $\left(EE\right)$ for both the uplink and downlink as shown in the following equations:

(1) $$Max\sum_{i=1,\;j=1}^{i=N,\;j=K}S_{ij\;}{}^{UL}S_{ij\;}{}^{UL}≔f_{1}\left(d_{CG},d_{ID},\;P_{C},\;P_{I}\right)$$

(2) $$Max\;\sum_{i=1,\;j=1}^{i=N,\;j=K}EE_{ij\;}{}^{UL}EE_{ij\;}{}^{UL}≔f_{2}\left(d_{CG},d_{ID},\;P_{C},\;P_{I}\right)$$

(3) $$Max\sum_{i=1,\;j=1}^{i=N,\;j=K}S_{ij\;}{}^{DL}S_{ij\;}{}^{DL}≔f_{1}\left(d_{BI},d_{GC},\;P_{B},\;P_{G}\right)$$

(4) $$Max\;\sum_{i=1,\;j=1}^{i=N,\;j=K}EE_{ij\;}{}^{DL}EE_{ij\;}{}^{DL}≔f_{2}\left(d_{BI},d_{GC},\;P_{B},\;P_{G}\right)$$

where $S_{ij\;}{}^{UL}$, $EE_{ij\;}{}^{UL}$, $S_{ij\;}{}^{DL}$ $\mathrm{and}\;EE_{ij\;}{}^{DL}$ are the overall system throughput and the total system energy efficiency for the uplink and downlink, respectively, of the *i*-th path between a CUE-D and the *j*-th path between IoTD and IoTG. Symbols $d_{CG}$, $d_{ID}$, $d_{BI}\;$, and $d_{GC}\;$are the uplink interference distance between CUE-IoTG and IoTD-D and the downlink interference distance between BS-IoTD and IoTG-CUE, respectively. Symbols $P_{C}$ and $P_{I}$ are the uplink CUE and IoTD transmission power, respectively. Symbols $P_{B}$ and $P_{G}$ are the downlink BS and IoTG transmission power, respectively.

For the proposed model, non-orthogonal multiple access (NOMA) was considered, NOMA can serve a large amount of devices and allow them to access the channel at the frequency/time and with the same transmission power [5,26]. Moreover, a Rayleigh fading channel with additive white Gaussian noise (AWGN) was considered for the proposed model [27]. Furthermore, for different links, it was assumed that the channel fading coefficient was statistically mutually independent.

#### 3.1.1. Uplink Data Communication

During the uplink data communication CUE transmits data to any destination whether it is BS or other CUEs. Furthermore, IoTD transmits its data to the IoTG. Thus, the received signal between the CUE-D ($r_{CD}$) and IoTD-IoTG ($r_{IG}$) links can be expressed as follows [28]:

(5) $$r_{CD}=\sqrt{P_{C}\;}H_{CD}\;X_{1}+\overset{K}{\underset{j=1}{\sum }}\sqrt{P_{Ij}\;}H_{IjD}\;Y_{1}+n_{1}$$

(6) $$r_{IG}=\sqrt{P_{I}\;}H_{IG}\;X_{2}+\overset{N}{\underset{i=1}{\sum }}\sqrt{P_{Ci}\;}H_{CiG}\;Y_{2}+n_{2}$$

where $H_{CD}$ and $X_{1}$ are the channel gain coefficient and the transmitted symbol of the CUE-D link, respectively. Symbol $P_{Ij}$ is the transmission power of the *j*-th IoTD. $H_{IjD}$ is the channel gain coefficient between IoTD-D. Symbol $Y_{1}$ represents the noise symbol received by D. Symbols $H_{IG}$ and $X_{2}$ are the channel gain coefficient and the transmitted symbol of the IoTD-IoTG link, respectively. Symbol $P_{Ci}$ is the transmission power of the *i*-th CUE. Symbol $H_{CiG}$ is the channel gain coefficient between CUE-IoTG. Symbol $Y_{2}\;$represents the noise symbol received by IoTG. Symbols$\;n_{1}$ and $\;n_{2}$ are the independent and identically distributed (i.i.d.) additive white Gaussian noise (AWGN) of the CUE-D and IoTD-IoTG, respectively. The signal-to-noise-plus- interference for CUE-D ($SINR_{thCD}$) and IoTD-IoTG ($SINR_{thIG}$) can be represented as follows:

(7) $$SINR_{thCD}=\frac{P_{C}\;H_{CD}}{\sum_{j=1}^{K}P_{Ij}\;H_{IjD}+N_{o}B}$$

(8) $$SINR_{thIG}=\frac{P_{I}\;H_{IG}}{\sum_{i=1}^{N}P_{Ci}\;H_{CiG}+N_{o}B}$$

where $N_{o}$ is the thermal noise power spectral density per Hertz. Symbol B is the channel system bandwidth. Symbols $H_{CD}$, $H_{IjD},\;H_{IG}$ and $H_{CiG}$ can be represented as:

(9) $$H_{CD}={\left|h_{CD}\right|}^{2}\gamma_{CD}$$

(10) $$H_{IjD}={\left|h_{IjD}\right|}^{2}\gamma_{IjD}$$

(11) $$H_{IG}={\left|h_{IG}\right|}^{2}\gamma_{IG}$$

(12) $$H_{CiG}={\left|h_{CiG}\right|}^{2}\gamma_{CiG}$$

where ${\left|h_{CD}\right|}^{2}$,$\;{\left|h_{IG}\right|}^{2}$, ${\left|h_{CiG}\right|}^{2}\mathrm{and}$ ${\left|h_{IjD}\right|}^{2}$ follow a complex normal distribution CN (0, 1). Symbols $\gamma_{CD}$_,_ $\gamma_{IG}$, $\gamma_{CiG}$_,_ and $\gamma_{IjD}$ represent the path loss model of CUE-D, IoTD-IoTG, *i*-th CUE-IoTG and the *j*-th IoTD-D, respectively. The path loss between CUE-D, IoTD-IoTG, *i*-th CUE-IoTG and the *j*-th IoTD-D can be expressed as [29,30]:

(13) $$\gamma_{CD}=\gamma_{o}\;d_{CD}{}^{-\alpha }$$

(14) $$\gamma_{IG}=\gamma_{o}\;d_{IG}{}^{-\alpha }$$

(15) $$\gamma_{CiG}=\gamma_{o}\;d_{CiG}{}^{-\alpha }$$

(16) $$\gamma_{IjD}=\gamma_{o}\;d_{IjD}{}^{-\alpha }$$

where $\gamma_{o}$ is the path loss constant of any transmission link symbol. Symbols $d_{CD}$ and $d_{IG}$ are the transmission distance between CUE-D and IoTD-IoTG, respectively. Symbol α is the path loss exponent. It is worth mentioning that the path loss will going to be changed based on the CUE communication with other CUEs or BSs. This is due to the difference between CUE-CUE or CUE-BS. Accordingly, Equations (7) and (8) can be written as:

(17) $$SINR_{thCD}=\frac{P_{C}\;\gamma_{o}\;d_{CD}{}^{-\alpha }}{\sum_{j=1}^{K}P_{Ij}\;\gamma_{o}\;d_{IjD}{}^{-\alpha }+N_{o}B}$$

(18) $$SINR_{thIG}=\frac{P_{I}\;\gamma_{o}\;d_{IG}{}^{-\alpha }}{\sum_{i=1}^{N}P_{Ci}\;\gamma_{o}\;d_{CiG}{}^{-\alpha }+N_{o}B}$$

Therefore, the overall system throughput (S) and the energy efficiency can be expressed as follows for the uplink data communication:

(19) $$S^{UP}=\overset{N}{\underset{i=1}{\sum }}log_{2}\left(1+SINR_{thCiD}\right)+\overset{K}{\underset{j=1}{\sum }}log_{2}\left(1+SINR_{thIjG}\right)$$

(20) $$EE^{UP}=\frac{\sum_{i=1}^{N}log_{2}\left(1+SINR_{thCiD}\right)}{\sum_{i=1}^{N}\left(P_{Ci}+P_{o}\right)}+\frac{\sum_{j=1}^{K}log_{2}\left(1+SINR_{thIjG}\right)}{\sum_{j=1}^{K}\left(P_{Ij}+P_{o}\right)}$$

where $P_{o}$ is the internal circuitry power.

Consequently, the objective function and constraints can be derived as:

(21) $$Max\;\sum_{i=1,\;j=1}^{i=N,\;j=K}S_{ij\;}{}^{UL}\;\mathrm{Subject}\;\mathrm{to}\;d_{CG}\geq d_{CGmin}\;d_{ID}\geq d_{IDmin}\;P_{C}\leq P_{Cmax}\;P_{I}\leq P_{Imax}$$

(22) $$\mathrm{and}\;Max\sum_{i=1,\;j=1}^{i=N,\;j=K}EE_{ij\;}{}^{UL}\;\mathrm{Subject}\;\mathrm{to}\;d_{CG}\geq d_{CGmin}\;d_{ID}\geq d_{IDmin}\;P_{C}\leq P_{Cmax}\;P_{I}\leq P_{Imax}$$

where $d_{CGmin}$ and $d_{IDmin}$ are the minimum required distance between CUE-IoTG and IoTG-D, respectively, for avoiding interference and enhancing the system performance. Symbols $P_{Cmax}$ and $P_{Imax}\;$ are the transmission power of the CUE and IoTD, which helps improving the system performance.

#### 3.1.2. Downlink Data Communication

During the downlink data communication BS and IoTG transmits their data to the *i*-th receiving CUE and *j*-th IoTD, respectively. Thus, the received signal between BS and any receiving CUE ($r_{BC}$) and IoTG and any receiving IoTD ($r_{GI}$) links can be expressed as follows [28]:

(23) $$r_{BC}=\sqrt{P_{B}\;}H_{BC}\;X_{3}+\sqrt{P_{G}\;}H_{GC}\;Y_{3}+n_{3}$$

(24) $$r_{GI}=\sqrt{P_{G}\;}H_{GI}\;X_{4}+\sqrt{P_{B}\;}H_{BI}\;Y_{4}+n_{4}$$

where $H_{BC}$ and $X_{3}$ are the channel gain coefficient and the transmitted symbol of the BS-CUE link, respectively. $H_{GC}$ is the channel gain coefficient between IoT and any receiving CUE. Symbol $Y_{3}$ represents the noise symbol received by any receiving CUE. Symbols $H_{GI}$ and $X_{4}$ are the channel gain coefficient and the transmitted symbol of the IoTG-IoTD link, respectively. Symbol $H_{BI}$ is the channel gain coefficient between BS-IoTD. Symbol $Y_{4}\;$represents the noise symbol received by IoTD. Symbols$\;n_{3}$ and$\;n_{4}$ are the independent and identically distributed (i.i.d.) additive white Gaussian noise (AWGN) of the BS-CUE and IoTG-IoTD, respectively. The signal-to-noise-plus- interference for BS-CUE ($SINR_{thBC}$) and IoTG-IoTD ($SINR_{thGI}$) can be represented as follows:

(25) $$SINR_{thBC}=\frac{P_{B}\;H_{BC}}{P_{G}\;H_{GC}+N_{o}B}$$

(26) $$SINR_{thGI}=\frac{P_{G}\;H_{GI}}{P_{B}\;H_{BI}+N_{o}B}$$

where $H_{BC}$, $H_{GC},\;H_{GI}$ and $H_{BI}$ can be represented as:

(27) $$H_{BC}={\left|h_{BC}\right|}^{2}\gamma_{BC}$$

(28) $$H_{GC}={\left|h_{GC}\right|}^{2}\gamma_{GC}$$

(29) $$H_{GI}={\left|h_{GI}\right|}^{2}\gamma_{GI}$$

(30) $$H_{BI}={\left|h_{BI}\right|}^{2}\gamma_{BI}$$

where ${\left|h_{BC}\right|}^{2}$,$\;{\left|h_{GC}\right|}^{2}$, ${\left|h_{GI}\right|}^{2}\mathrm{and}$ ${\left|h_{BI}\right|}^{2}$ follow a complex normal distribution CN (0, 1). Symbols $\gamma_{BC}$_,_ $\gamma_{GC}$, $\gamma_{GI}$_,_ and $\gamma_{BI}$ represent the path loss model of BS-CUE, IoTG-CUE, IoTG-IoTD and BS-IoTD, respectively. Thus, the path loss between BS-CUE, IoTG-CUE, IoTG-IoTD and BS-IoTD can be expressed as [29,30]:

(31) $$\gamma_{BC}=\gamma_{o}\;d_{BC}{}^{-\alpha }$$

(32) $$\gamma_{GC}=\gamma_{o}\;d_{GC}{}^{-\alpha }$$

(33) $$\gamma_{GI}=\gamma_{o}\;d_{GI}{}^{-\alpha }$$

(34) $$\gamma_{BI}=\gamma_{o}\;d_{BI}{}^{-\alpha }$$

where $d_{BC}$ and $d_{GI}$ are the transmission distance between BS-CUE and IoTG-IoTD, respectively. Therefore, Equations (25) and (26) can be written as:

(35) $$SINR_{thBC}=\frac{P_{B}\;\gamma_{o}\;d_{BC}{}^{-\alpha }}{P_{G}\;\gamma_{o}\;d_{GC}{}^{-\alpha }+N_{o}B}$$

(36) $$SINR_{thGI}=\frac{P_{G}\;\gamma_{o}\;d_{GI}{}^{-\alpha }}{P_{B}\;\gamma_{o}\;d_{BI}{}^{-\alpha }+N_{o}B}$$

Therefore, the overall system throughput (S) and the energy efficiency can be expressed as follows for the downlink data communication:

(37) $$S^{DL}=\overset{N}{\underset{i=1}{\sum }}log_{2}\left(1+SINR_{thDCi}\right)+\overset{K}{\underset{j=1}{\sum }}log_{2}\left(1+SINR_{thGIj}\right)$$

(38) $$EE^{DL}=\frac{\sum_{i=1}^{N}log_{2}\left(1+SINR_{thDCi}\right)}{\left(P_{B}+P_{o}\right)}+\frac{\sum_{j=1}^{K}log_{2}\left(1+SINR_{thGIj}\right)}{\left(P_{G}+P_{o}\right)}$$

Consequently, the objective function and constraints can be derived as:

(39) $$Max\;\sum_{i=1,\;j=1}^{i=N,\;j=K}S_{ij\;}{}^{DL}\;\mathrm{Subject}\;\mathrm{to}\;d_{BI}\geq d_{BImin}\;d_{GC}\geq d_{GCmin}\;P_{B}\leq P_{Bmax}\;P_{G}\leq P_{Gmax}$$

(40) $$\mathrm{and}\;Max\sum_{i=1,\;j=1}^{i=N,\;j=K}EE_{ij\;}{}^{DL}\;\mathrm{Subject}\;\mathrm{to}\;d_{BI}\geq d_{BImin}\;d_{GC}\geq d_{GCmin}\;P_{B}\leq P_{Bmax}\;P_{G}\leq P_{Gmax}$$

where $d_{BImin}$ and $d_{GCmin}$ are the minimum required distance between BS-IoTD and IoTG-CUE, respectively, for avoiding interference and enhancing the system performance. Symbols $P_{Bmax}$ and $P_{Gmax}\;$ are the maximum transmission power of BS and IoTG, which helps improve the system performance.

For the proposed model, for fairness, it is assumed that the required signal-interference-plus-noise for uplink and downlink for a 5G network (*SINR_thCiB_*_,_ *SINR_thBCi_*) and IoT system (*SINR_thIjG_*, *SINR_thGIj_*) has the same value, which is *SINR_th_*_._

### 3.2. Dataset Generation

The datasets used in this work were generated using MATLAB simulations based on the equations previously explained in Section 3.1.1 for the uplink and Section 3.1.2 for the downlink communication. The parameters in the equations were substituted for by the values declared in Table 1. Two datasets were generated, one for the uplink and the other for the downlink communication. The datasets will be used to train a model that is to be placed on all sending devices in the case of uplink and another to be placed on BS and IoTG in the case of downlink communication.

For the uplink communication, the outputs were generated for different combinations of *SINR_th_* and distances of CUE-D and IoTD-IoTG using the Lagrange optimization technique to generate the optimal distances of IoTD-D and CUE-IoTG for each input. The experiments were run for different values of *SINR_th_* ranging from 0 to 20. For each value of *SINR_th_*, the value of the CUE-D distance was initialized to 1 and incremented by half a meter for each record until the throughput and energy efficiency of the calculated distances of IoTD-D and CUE-IoTG were unacceptable.

Considering the downlink communication, the output distances IoTG-CUE and BS-IoTD were generated for different values of *SINR_th_*_,_ along with distances of BS-CUE and IoTG-IoTD. The output distances were evaluated for each record to make sure that they meet the required throughput and energy efficiency.

**Figure 2:**
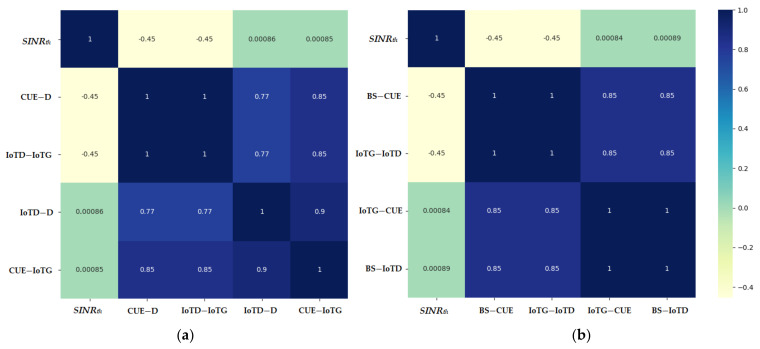
Spearman correlation of all features generated for (**a**) the uplink dataset and (**b**) the downlink dataset.

The statistical description of all features in the two generated datasets showing the minimum, maximum, mean, standard deviation and total number of records are shown in Table 2 and Table 3. The spearman correlation of all input and output features for both the uplink and downlink communication was calculated and is presented in Figure 2. In the results section, the effect of the correlation will be further explained.

### 3.3. Proposed Deep Learning Model

One of the main deep learning architectures most commonly used is the convolutional neural networks (CNN), which has become almost a standard in a variety of two-dimensional (2D) data applications, especially image and video processing. Recently, a modification of the traditional 2D-CNN, namely the 1D-CNN, was proposed in [35] and later showed outstanding performance in numerous studies given a limited amount of signal data. Examples of such applications are biomedical classification, speech recognition, fault detection in a motor and several others [36].

There are several advantages that could be achieved when using 1D-CNN. Compared to ordinary deep learning methods, it has proved to generate good results even if the training records are scarce. 1D-CNN has a low computational complexity, making it much easier and faster to train. It is very well suited for being used in real-time applications on mobile devices as they consume minimal processing and battery power [36]. Consequently, 1D-CNN was deployed in this research work to be the feature extraction methodology for the proposed model. In this section, the proposed deep learning model is introduced. The presented model is to be implemented on sending devices, BS and IoTG separately to calculate the optimal distance required to reduce the interference.

#### 3.3.1. Network Structure

In this work, a distributed deep learning network is proposed having two sub-models each comprising the input, 1D CNN, and fully connected and output layers. The proposed trained network is intended to be used by each device independently thus predicting the optimal distance for minimal interference and therefore the best throughput and energy efficiency. Two models are to be trained, one for uplink communication and the other for downlink communication, each using one of the generated datasets.

**Figure 3:**
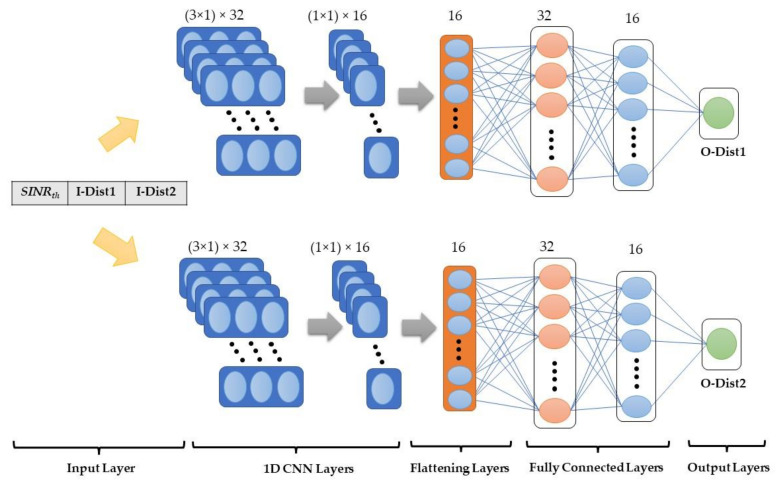
Proposed deep learning network that is to be used by each device independently to calculate optimal output distances.

The choice of the number of hidden layers was based on multiple experiments leading to the network depicted in Figure 3. The figure shows the input at a single time to the proposed model knowing that any device using this model will need to input new entries each time it needs to calculate the optimal distance to avoid interference. Each sub-model is inputs the values of *SINR_th_*, input distance 1 (I-Dist1) and input distance 2 (I-Dist2). I-Dist1 represent the CUE-D for uplink communication and BS-CUE for downlink communication while I-Dist2 denotes IoTD-IoTG for uplink communication and IoTG-IoTD for communication.

Each sub-model is then trained independently to learn to predict one of the output distances: O-Dist1 and O-Dist2. O-Dist1 represents the IOTD-D for uplink and IoTG-CUE for downlink while O-Dist2 represents the CUE-IOTG and BS-IoTD for downlink. The values are input to the 1D-CNN layers for feature extraction and followed by fully connected layers for calculating the estimated distances as a regression problem. For each sub-model, the layers are defined as follows:

- An abstract input layer that takes the current values of the input and passes it to the 1D-CNN layers
- The first 1D-CNN is 3 × 1 having 32 filters, with a kernel size of 3
- The second 1D-CNN is 1 × 1 having 16 filters, with a kernel size of 1
- A flattening layer to reshape the 1D CNN can be input to the fully connected layers
- A 32-neuron fully connected layer
- A 16-neuron fully connected layer
- An output layer to produce the regression distance result

#### 3.3.2. Data Scaling

To achieve the best results in learning the deep learning network parameters, data must be normalized. In this work the data were normalized using the min-Max normalization, which applies the following equation to each feature

(41) $$x_{scaled}=\frac{x-x_{min}}{x_{max}-x_{min}}$$

where x represents the data to be normalized, $x_{min}$ is the minimum value, $x_{max}$ is the maximum value in the feature addressed, and $x_{scaled}$ is the output normalized value ranging from 0 to 1.

#### 3.3.3. Activation Function

Since the presented network aims to predict the distance between devices, it cannot have a negative output. This led to the choice of the Parametric Rectified Linear Unit (PReLU) activation function, which was first proposed in [37] as a generalization of the traditional Rectified Linear Unit (ReLU), which applies the equation:

(42) $$F(w_{i})=\left\{\begin{array}{c} w_{i},\;\;\;\;\;\;\;if\;w_{i}>0\\ a_{i}w_{i}\;\;\;\;\;\;if\;w_{i}\leq 0 \end{array}\right.$$

where $F(w_{i})$ represents the output of the activation function, $w_{i}$ represents the *i*th input and $a_{i}$ is the *i*th alpha. PReLU manages to adaptively learn the suitable alpha to fit values below zero propagating through the network from the training data.

#### 3.3.4. Optimization Function

The optimization method applied in this network is the adaptive moment estimation (Adam), which was proposed in [38] as an improvement to the stochastic gradient descent (SGD) since it adaptively handles gradients in sparse data. The loss function chosen with Adam was the mean absolute error as it gives a real estimate of how far the average distance prediction is from the actual data, thus helping the network minimize it.

#### 3.3.5. Parameter Optimization

Optimum parameter choice was based on the grid search function using different values of batch sizes [64,128,256,512] and epochs [50,100,150,200,250] for both sub-models. The parameters that were most suitable to both sub-models were a batch size of 128 and 100 epochs, but the experiments were performed for 200 epochs to ensure the models learned sufficiently.

## 4. Results and Discussion

In this section, the performance evaluation of the deep learning architecture when compared to selected benchmarks is presented. Furthermore, the performance of the proposed approach was examined in terms of optimized energy efficiency, and optimized overall system throughput through MATLAB and Python simulations.

### 4.1. Deep Learning Model Results Evaluation

In order to evaluate the goodness of fit of the proposed model, 10-fold cross validation is used to compare the average results produced by the proposed model to those of other benchmarks based on the following metrics:

- Mean Absolute Error (MAE), which measures the average differences between actual and predicted values.

(43) $$MAE=\frac{1}{n}\;\overset{n}{\underset{i=1}{\sum }}\left|y_{i}-{\hat{y}}_{i}\right|$$

- Root Mean Squared Error, which calculates the square root of the average of the squared differences between actual and predicted values as

(44) $$RMSE=\sqrt{\frac{1}{n}\;\overset{n}{\underset{i=1}{\sum }}{\left(y_{i}-{\hat{y}}_{i}\right)}^{2}}$$

given that the number of records in the test subset is represented by n, $y_{i}$ is the actual value, and ${\hat{y}}_{i}$ is the predicted value.

Multiple grid search experiments were performed to obtain optimal parameters for the models used in the benchmark comparisons. Table 4 and Table 5 show the generated optimal parameters for each benchmark using both the uplink and downlink datasets, respectively. The benchmarks used in our comparisons were the support vector regressor, random forest regressor, Adaboost regressor and multilayer perceptron.

Table 6 shows the average of all folds’ results when comparing the trained uplink model to support the vector regressor, random forest regressor, Adaboost regressor, and multilayer perceptron using their optimal parameters. The results show that the random forest regressor tended to produce an overfitted model as the training error was much lower than the testing error. The proposed model outperformed all the other methods in testing while maintaining a small difference from the training error thus showing no signs of overfitting. It can be noted that the results produced for the IoT-D distance tended to have a higher error that that of the CUE-IoTG in all the models. This can be related to the correlation presented in Section 3.2 where the correlation of the of the IoT-D with the inputs was lower than that of the CUE-IoTG making it harder to predict.

Table 7 shows the same comparison but using the downlink dataset to calculate the distances IoTG-CUE and BS-IoTD that minimize interference. It can be deduced from the results that the proposed model in this comparison resembles the performance of the support vector regressor, random forest regressor, and the multilayer perceptron when applying the optimal parameters previously declared in Table 5.

**Figure 4:**
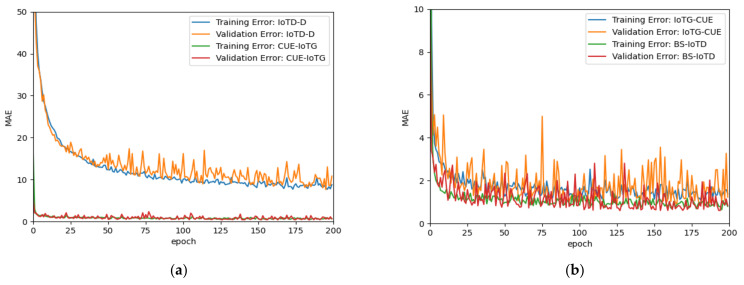
Mean absolute error generated by the training and validation data when calculating the output distances O-Dist1 and O-Dist2 for: (**a**) uplink communication dataset and (**b**) downlink communication dataset.

Another experiment involved splitting the dataset into a two-third training and one-third testing schema to train a single network to further analyze the results. The training data were used to build the model while keeping 20% for validation. Figure 4a,b shows the mean absolute error produced while training and validating for both models. Both figures show that after epoch 100, the results were hardly changing, thus not requiring any further training. It can be noted from the figures that the models were not overfitted since the training and validating errors were around the same values for each output independently.

### 4.2. Analytical Evaluation

In this section, further analytical evaluation of the results obtained when splitting the dataset into two-thirds training and one-third testing was performed. The records used for training the model were not used in the testing phase to assure that the analysis of results was not over-rated by data that the model had already learned from. The conditions for predicting the optimal required distance for controlling interference were revealed by analyzing the results obtained. Same experimental assumptions as in [14] were considered. The network parameters considered for simulation are listed in Table 1.

**Figure 5:**
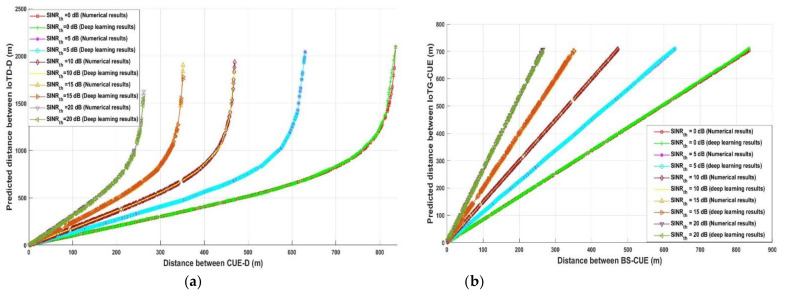
(**a**) Distance between CUE-D versus predicted distance between IoTD-D (uplink) (**b**) Distance between BS-CUE versus predicted distance between IoTG-CUE (downlink).

Figure 5 depicts the predicted required distance between any IoTD-D link for the uplink data communication and between BS-IoTD for downlink data communication with different values of *SINR_th_* for the proposed model using the analytical and deep learning model. For the uplink data communication, it was assumed that all the transmitted devices, whether they were CUEs or IoTDs, always had a maximum transmission power equal to 23 dBm. Thus, it can be noticed from Figure 5a, for the analytical and deep learning model, the optimum required distance between IoTD-D (*d_ID_*) to decrease the interference at the destination increased when the distance between CUE-D (*d_CD_*) increased. In addition, it can be mentioned that in order for *d_CD_* to reach the maximum value, *SINR_th_* decreased gradually since increasing the transmission distance led to increasing the losses in the communication link. Consequently, decreasing transmission distance increased *SINR_th_* —for example, to have a communication link with *SINR_th_* equal to 0 dB the transmission distance *d_CD_* remained effective until it exceeded 836 m. Additionally, when *d_CD_* was 600.5 m, *d_ID_* must be greater or equal to 647.33 m analytically and 646.8 m using the deep learning model. On the other hand, when the required *SINR_th_* for any communication link was 20 dB, the maximum transmission distance for reaching effective communication was 261.5 m. It can also be noticed that, the required distance between IoTD-D was equal to 317.2 and 316.72 assuming that *d_CD_* was 99.64 m, using analytical and deep learning model, respectively.

For the downlink data communication, the transmission power of BS and IoTG was 46 dBm and 43 dBm, respectively, which is considered high when comparing it with the CUE and IoTD transmission power. Therefore, it can be observed from Figure 5b that increasing the required *SINR_th_* led to an increase in the required distance between IoTG-CUE to avoid interference. For example, when *SINR_th_* was 0 dB and the downlink transmission distance (*d_BC_*) was 600.5 m, the required distance between IoTG-CUE (*d_GC_*) to avoid interference was 505.52 m numerically and 506.95 using deep learning, while when *SINR_th_* was 5 dB, *d_GC_* must be 677.5 m and 677.8 m for numerical and deep learning, respectively. On the other hand, when *SINR_th_* was 20 dB and *d_BC_* was 99.5 it can be found that *d_GC_* must be in the range of 265 m to avoid interference.

It is worth mentioning from Figure 5 that when comparing the results of the uplink and downlink, the highest transmission power of BS and IoTG and the shortest distance between IoTD-IoTG led to decreased interference at its destination. On the other hand, decreasing the transmission power led to increasing interference, which required an increase in the distance between any interference device and transmission link.

**Figure 6:**
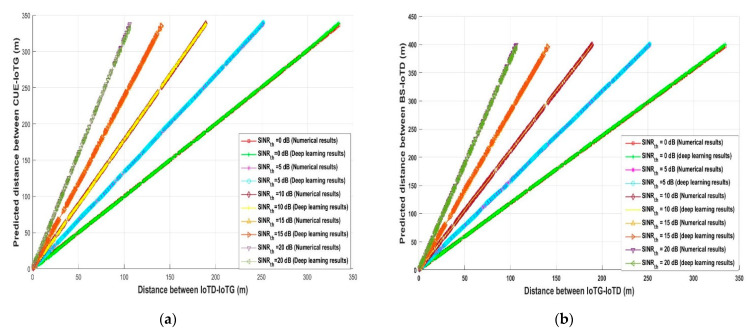
(**a**) Distance between IoTD-IoTG versus predicted distance between CUE-IoTG (uplink) (**b**) Distance between IoTG-IoTD versus predicted distance between BS-IoTD.

The same result was obtained when the IoT system performance is evaluated through uplink and downlink data communication, as demonstrated in Figure 6, to decrease the interference at IoTG (uplink) and IoTD (downlink). For increasing the system reliability, the distance between any transmission CUE and IoTG for the uplink (*d_CG_*) and for downlink (*d_GC_*) must be greater than the distance between IoTD and IoTG for uplink (*d_IG_*) and for downlink (*d_GI_*) —for example, as shown in Figure 6a in the case of uplink, when dIG was equal to 240.2 m; the distance dCG should be 240.6 m analytically and 241 m using deep learning when the required SINRth is 0 dB. While, when dIG was equal to 44 m and *SINR_th_* was 20 dB, *d_CG_* was 139.17 m analytically and 139.63 m using deep learning. On the other hand, in case of the downlink as demonstrated in Figure 6b, when the required SINRth was 0 dB and dGI was equal to 240.2 m the distance *d_GC_* should be 285.48 m analytically and 286.35 m using deep learning. While, when *d_GI_* was equal to 44 m, *d_GC_* should be 165.37 m analytically and 165.33 m using deep learning when *SINR_th_* was 20 dB. Additionally, it can be noticed that decreasing the distance between any source and destination links leads to decreasing the path loss and increasing the *SINR_th_*. Based on the proposed model, it is assumed that the transmission distance between CUE-BS (uplink) and BS-CUE (downlink) is greater than the transmission distance between IoTD-IoTG (uplink) and IoTG-IoTD (downlink). It has been concluded from Figure 6 that, the interference distance (*d_GC_*) during the downlink must be greater than the interference distance (*d_CG_*) during the uplink, this due to the highest transmission power of IoTG and the nearest distance between IoTG-IoTD, which increases the interference at CUE. That is why the interference distance during the downlink (*d_GC_*) should increase compared with the interference distance during the uplink (*d_GC_*).

**Figure 7:**
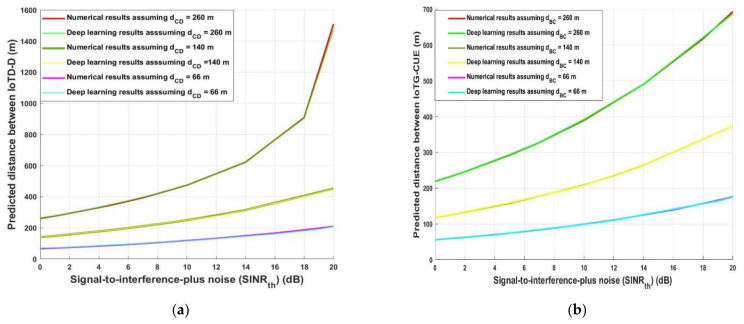
(**a**) Signal-to-interference-ratio- plus-noise (*SINR_th_*) versus predicted distance between IoTD-D (uplink) (**b**) Signal-to-interference-ratio- plus-noise (*SINR_th_*) versus predicted distance between IoTG-CUE (dowlink).

The predicted required *d_ID_* and *d_GC_* distance for decreasing the interference at any D (uplink) and decreasing the interference at CUE (downlink) was examined again in Figure 7 for the analytical and deep learning model for different transmission distances and against *SINR_th_*. Different uplink (*d_CD_*) and downlink transmission (*d_BC_*) distance values were assumed such as 66, 140, and 260 m and against *SINR_th_*, which varied from 0 to 20 dB to predict *d_ID_* and *d_BI_*. As can be observed, when *SINR_th_* increases the predicted required uplink *d_ID_* and downlink *d_BI_* must be greater than or equal to uplink *d_CD_* and downlink *d_BC_*, respectively, for decreasing the interference and at the same time satisfying the system requirements in term of *SINR_th_*—for example, as shown in Figure 7a when *d_CD_* was 260 m, the optimum required *d_ID_* was 260.6 m analytically and 264.24 using deep learning when *SINR_th_* = 0 dB, while when *SINR_th_* = 18 dB for the same distance *d_CD_*, the predicted required distance *d_ID_* was 907.72 m analytically and 903.74 using deep learning. On the other hand, when *d_ID_* was 66 m and *SINR_th_* = 4 dB, *d_CD_* must be 83.09 and 85.75 analytically and based on deep learning, respectively. Furthermore, for the downlink data communication as shown in Figure 7b, for *SINR_th_* = 0 dB and *d_BC_* = 260 m, the required distance to avoid interference *d_GC_* was 218.77m and 219.24 using analytical and the deep learning algorithm, respectively, while for the same transmission distance *d_GC_* should be in the range of 619 m to avoid interference and fulfil the required *SINR_th_*, which is 18 dB. Additionally, when *d_BC_* was 66 m, the required distance for avoiding interference should be in the range of 55.6 m if *SINR_th_* is 0 dB and 176 m when *SINR_th_* = 20 dB.

**Figure 8:**
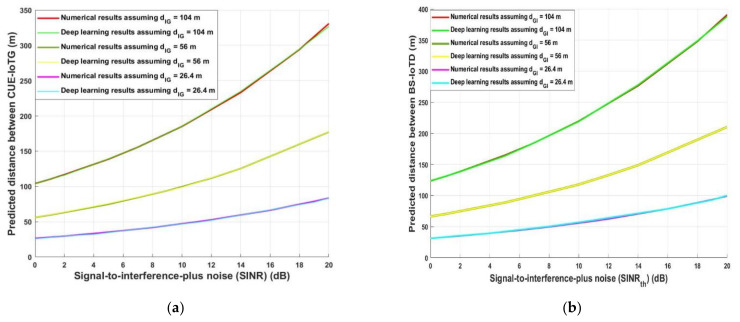
(**a**) Signal-to-interference-ratio-plus-noise (*SINR_th_*) versus predicted distance between CUE-IoTG (uplink) (**b**) Signal-to-interference-ratio-plus-noise (*SINR_th_*) versus predicted distance between BS-IoTD (downlink).

Furthermore, Figure 8 demonstrates the predicted required uplink and downlink distances (*d_CG_* and *d_BI_*) for decreasing the interference at IoTG (uplink) and IoTD (downlink), respectively. A different scenario is proposed to evaluate the system performance for the uplink and downlink data communication, assuming that *d_IG_* and *d_GI_* are 104 m, 56 m, and 26.4 m for different values of *SINR_th_*. As shown in Figure 8a, in case of uplink, when the required *SINR_th_* increased, for different transmission *d_IG_*, the distance between *d_CG_* increased—for example, when *d_IG_* was equal to 104 m and *SINR_th_* was equal to 7 dB, the optimum required *d_CG_* for the analytical and deep learning model was 155.65 m and 155.54 m, respectively. However, for the same transmission distance when *SINR_th_* was equal to 18 dB, *d_CG_* was 294.2 m analytically and 294.46 m using the deep learning model. The same performance was obtained when the system was evaluated during the downlink as shown in Figure 8b, e.g., when *SINR_th_* was equal to 7 dB and *d_GI_* was equal to 104 m and, the optimum required *d_CG_* for the analytical and deep learning model was 185 m. However, for the same transmission distance *d_CG_* is 348 m and 348.86 m for the analytically and deep learning model, respectively, when *SINR_th_* was equal to 18 dB. Figure 7 and Figure 8 show that the required *SINR_th_* is an important parameter that should affect the predicted required interference distance for decreasing the interference at any destination. Additionally, using these results and based on the system requirements and environmental conditions, an adaptive smart system should be engaged to enhance the system performance for both CUE and IoT networks.

**Figure 9:**
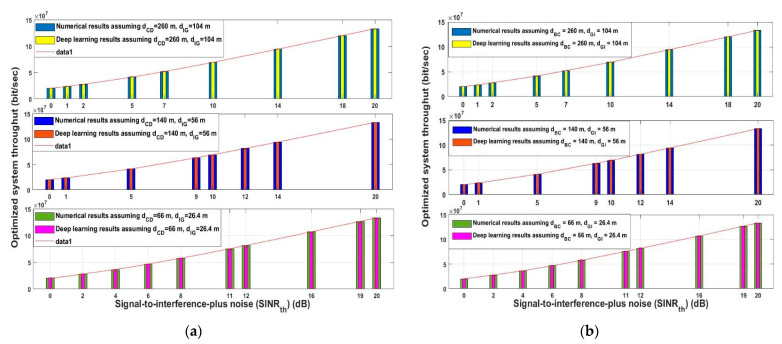
Signal-to-interference-ratio-plus-noise (*SINR_th_*) versus optimized system throughput (**a**) uplink (**b**) downlink.

**Figure 10:**
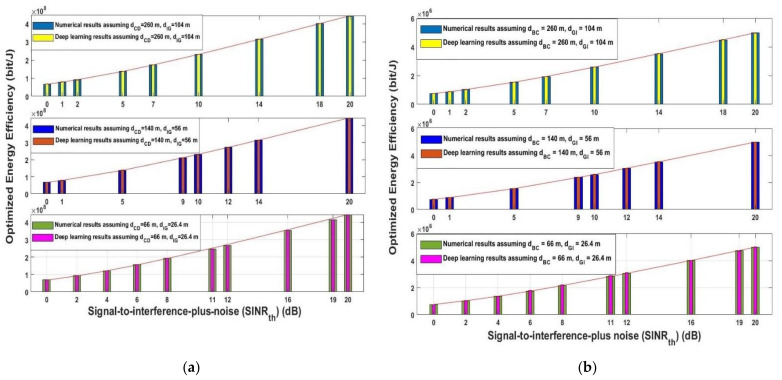
Signal-to-interference-ratio-plus-noise (*SINR_th_*) versus optimized energy efficiency (**a**) uplink (**b**) downlink.

As mentioned earlier, since the dataset used in the analysis was split into two-thirds training and one-third testing, the results shown in Figure 9 and Figure 10 are based only on records available in the testing data. As a result, some of the *SINR_th_* values did not exist in testing records. Figure 9 demonstrates the optimized system throughput for the proposed approach for the uplink and downlink data communication using the analytical and deep learning model for different randomly chosen *SINR_th_* values. For fair performance evaluation three different scenarios were considered. Assuming that the distance combinations between the uplink distances (*d_CD_* and *d_IG_*) and the downlink distances (*d_BC_* and *d_GI_*) were considered to be the same; they were 260 m and 104 m, 140 m and 56 m, and 66 m and 26.4 m, respectively. The chosen distances represented long, intermediate, and short distances. As observed for the uplink and downlink data communication represented in Figure 9a,b, respectively, for the three different scenarios when the *SINR_th_* increased, the optimized system throughput increased. Additionally, it can be noticed that for the three scenarios for any *SINR_th_*, the optimized system throughput value was approximately identical for the analytical and deep learning models. This means that the proposed approach is capable of reaching the maximum system throughput regard less of the transmission was (long-intermediate-short), as the aim of the proposed model is to predict the interference transmission distance between any interfering node and any destination, for trying to prevent interference and increase system reliability.

The same performance was obtained when the optimized energy efficiency for the uplink and downlink was examined for the assumed three different scenarios in Figure 10. As depicted from Figure 10a uplink data communication and Figure 10b downlink data communication, the optimized energy efficiency always increased with the increased of *SINR_th_* for the analytical and deep learning models. The proposed model succeeded in keeping the optimized energy efficiency approximately the same for the three different assumed transmission distances. Figure 10 is correlated with the results obtained in Figure 9. These two results show the effectiveness of the proposed model in predicting the position of the interference nodes, as by knowing the distance between them and any destination helps prevent the interference, thus increasing the system performance.

**Figure 11:**
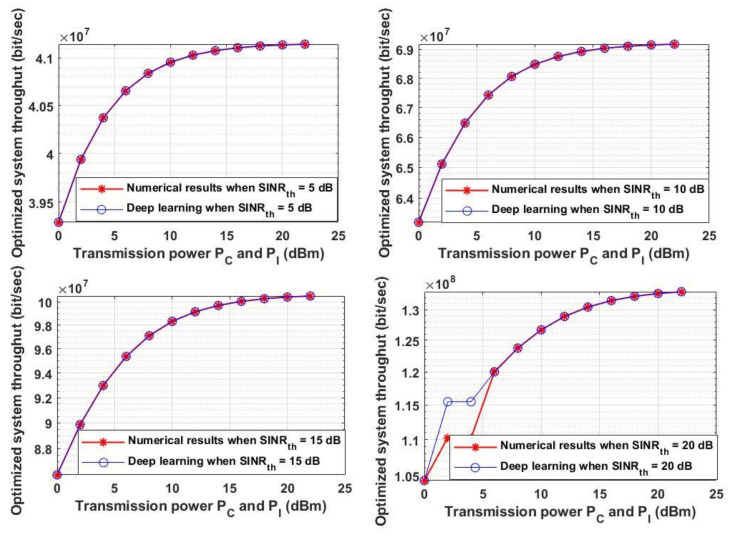
Transmission power P_C_ and P_I_ versus optimized system throughput.

As an extra assessment of the proposed model, the distances obtained from both the analytical and deep learning models were both input to Equations (20) and (21) to calculate the throughput and energy efficiency for different values of transmission power. The optimized system throughput for the proposed approach was evaluated once again in Figure 11 for four different randomly chosen *SINR_th_* values with different transmission powers for CUE (*P_C_*) and IoTD (*P_I_*). It was assumed that the values of *SINR_th_* were 5, 10, 15 and 20 dB, respectively. As depicted in Figure 11, for any *SINR_th_* increasing the transmission power leads to increasing the system throughput for the analytical and deep learning model. As the system is always limited by channel noise, pathloss and interference that is why the transmission power is one of the parameters, which can overcome the channel conditions. Thus, increasing or decreasing the transmission power must be considered according to the channel conditions and the required system QoS. Furthermore, by comparing the four different *SINR_th_* values it can be found that increasing the *SINR_th_* increases the overall system throughput, which is correlated with the results obtained in Figure 9.

**Figure 12:**
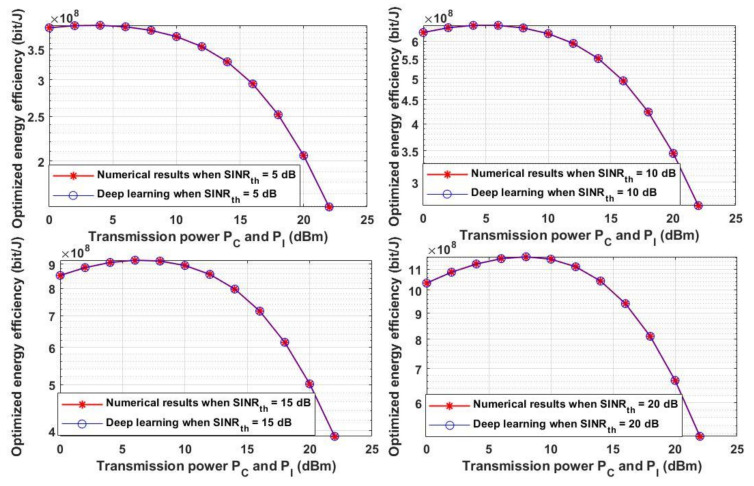
Transmission power P_C_ and P_I_ versus optimized energy efficiency.

Moreover, the optimized energy efficiency is analyzed in Figure 12 for the same chosen *SINR_th_* stated in Figure 9 and with different *P_C_* and *P_I_*. As shown in Figure 12 each value of *SINR_th_* yields a maximum transmission power that leads to an optimum energy efficiency—for example, when *SINR_th_* = 0 dB, the maximum transmission power for any sender node to reach the optimum energy efficiency is 2 or 4 dBm, while when *SINR_th_* = 5 dB, the maximum power is 4 or 6 dBm to reach the maximum energy efficiency. On the other hand, when *SINR_th_* is 20 dB, the maximum transmission power is energy efficiency is 8 dBm. It can be deduced from this figure that increasing the transmission power may lead to a decrease in the energy efficiency as the increment of the transmission power incr11eases the system cost and decreases the system energy efficiency. By comparing Figure 11 and Figure 12, increasing the transmission power increases the overall system throughput and at the same time may decreases the energy efficiency. Thus, for obtaining the maximum system throughput with the highest energy efficiency, the two performances can be jointly considered in order to obtain the required system performance based on the two metrics.

## 5. Conclusions

A novel interference avoidance system was proposed for a 5G network and IoT using analytical and deep learning techniques. First an analytical model was created and simulated using MATLAB to calculate the optimal distances required between IoTD and D. In addition, the model calculated the optimal distance between CUE and IoTG. A deep learning model was then proposed that adopted the 1D-CNN. 1D-CNN was recently introduced and has been proven to have low computational complexity, and thus managing to conserve processing and battery, which make it very suited to be deployed in devices in real-time applications. Consequently, the deep learning model on CUE and IoTD for uplink and on BS and IoTG for downlink could generate the appropriate interference distance to meet the near-optimal result. This model was assessed by a 10-fold cross validation data split using data generated from the MATLAB simulations and produced very low mean absolute error and root mean square error when compared to various benchmarks. Next, the analytics of the results of predicting the minimum acceptable interference distance between IoTD-D and CUE-IoTG for uplink and the distance between BS-IoTD and IoTG-CUE for downlink resulting in achieving near-optimal throughput and energy efficiency were demonstrated. Based on the results obtained in terms of system throughput and energy efficiency, it has been shown that the proposed model can exhibit the best performance under different environmental conditions. The problem of interference has been discussed and solved using the Lagrange optimization technique and deep learning. Both techniques have been used to predict the optimum interference distance between CUE-IoTG and IoTD-D for uplink and the optimum interference distance between IoTG-CUE and BS-IoTD for downlink. Additionally, based on the analytical and deep learning, it has been proven that the interference distance must be greater than the transmission distance between the CUE-D and IoTD-IoTG links to avoid or decrease the interference among any destination (BS or CUE receiver). In addition, it has been shown how during the downlink data communication, the high BS and IoTG lead to a decrease the interference distance, as increasing the transmission power leads to overcoming the interference at any communication link. Furthermore, the effect of *SINR_th_* and the transmission power on predicting the maximum required interference distance was investigated. It was shown that increasing *SINR_th_* leads to increasing the interference distance between CUE-IoTG, IoTD-D, IoTG-CUE and BS-IoTD. Moreover, it has been shown that increasing the transmission power increases the overall system performance. Additionally, among different values of transmission power, one can reach the maximum energy efficiency. The obtained results show that the proposed model can achieve the maximum system throughput and energy efficiency with suitable system reliability.

## Figures and Tables

**Table 1:** Simulation Parameters.

Parameters	Value
$N_{o}$	174 dBm [31]
B	10 MHz
*SINR_th_*	20 dB [32]
*P_c_*	23 dBm [32]
*P_I_*	23 dBm [32]
*P_B_*	46 dBm [9,25]
*P_G_*	43 dBm [33,34]
α	4
γ_o_	10^−1^ [32]
*f_c_*	2 GHz

**Table 2:** Statistical description of features in the generated uplink dataset.

	*SINR_th_*	CUE-D	IoTD-IoTG	IoTD-D	CUE-IoTG
Number of records	21,055	21,055	21,055	21,055	21,055
Minimum	0.00	1.00	0.40	1.00	0.40
Maximum	20.00	840.00	336.00	4644.00	338.65
Mean	7.94	281.23	112.49	501.97	168.77
Standard Deviation	5.84	190.82	76.33	395.39	97.39

**Table 3:** Statistical description of features in the generated Downlink dataset.

	*SINR_th_*	BS-CUE	IoTG-IoTD	IoTG-CUE	BS-IoTD
Number of records	21,055	21,055	21,055	21,055	21,055
Minimum	0	1	0.4	0.84	0.48
Maximum	20	840	336	709	400
Mean	7.94	281.23	112.49	354.39	200.15
Standard Deviation	5.84	190.82	76.33	204.09	115.22

**Table 4:** Optimal parameters generated for the benchmarks used in the uplink model evaluation.

Benchmarks	IoTG-CUE	BS-IoTD
Support vector regressor	kernel = ‘rbf’, C = 220, gamma = 40	Kernel = ‘rbf’, C = 200, gamma = 50
Random forest regressor	max_depth = 100, max_features = 3, min_samples_leaf = 3, min_samples_split = 8, n_estimators = 1000	max_depth = 90, max_features = 3, min_samples_leaf = 3, min_samples_split = 8, n_estimators = 1000
Adaboost regressor	learning_rate = 0.01, loss = ‘Linear’, n_estimators = 150	learning_rate = 1, loss = ‘linear’, n_estimators = 150
Multilayer perceptron	activation = ‘tanh’, alpha = 0.05, solver = ‘sgd’, hidden_layer_sizes = (300,), learning_rate = ‘adaptive’	activation = ‘tanh’, alpha = 0.05, solver = ‘sgd’, hidden_layer_sizes = (300,), learning_rate = ‘adaptive’

**Table 5:** Optimal parameters generated for the benchmarks used in the downlink model evaluation.

Benchmarks	IoTG-CUE	BS-IoTD
Support vector regressor	kernel = ‘rbf’, C = 220, gamma = 40	Kernel = ‘rbf’, C = 200, gamma = 50
Random forest regressor	max_depth = 100, max_features = 3, min_samples_leaf = 3, min_samples_split = 8, n_estimators = 1000	max_depth = 90, max_features = 3, min_samples_leaf = 3, min_samples_split = 8, n_estimators = 1000
Adaboost regressor	learning_rate = 0.1, loss = ‘square’, n_estimators = 100	learning_rate = 1, loss = ‘linear’, n_estimators = 100
Multilayer perceptron	activation = ‘tanh’, alpha = 0.05, solver = ‘sgd’, hidden_layer_sizes = (100,), learning_rate = ‘adaptive’	activation = ‘tanh’, alpha = 0.05, solver = sgd, hidden_layer_sizes = (100,), learning_rate = ‘adaptive‘

**Table 6:** Average result of the 10-fold cross validation method comparing the proposed uplink model versus various benchmarks including the support vector regressor, random forest regressor, Adaboost regressor, and multilayer perceptron.

	IoTD-D				CUE-IoTG			
	MAE		RMSE		MAE		RMSE	
Benchmarks	Train	Test	Train	Test	Train	Test	Train	Test
Support vector regressor	12.83	15.14	96.29	94.28	0.07	0.75	0.07	1.14
Random forest regressor	2.52	11.63	35.32	64.84	0.11	0.83	0.18	1.16
Adaboost regressor	128.06	129.21	215.24	216.70	18.13	18.36	21.69	21.90
Multilayer perceptron	21.86	24.64	77.00	80.97	0.16	0.78	0.26	1.16
Proposed model	9.59	9.84	66.09	63.43	0.77	0.77	1.01	1.06

**Table 7:** Average result of the 10-fold cross validation method comparing the proposed downlink model versus various benchmarks including the support vector regressor, random forest regressor, Adaboost regressor, and multilayer perceptron.

	IoTG-CUE				BS-IoTD			
	MAE		RMSE		MAE		RMSE	
Benchmarks	Train	Test	Train	Test	Train	Test	Train	Test
Support vector regressor	0.17	1.56	0.24	2.37	0.14	0.89	0.20	1.34
Random forest regressor	0.26	1.74	0.39	2.43	0.16	0.98	0.24	1.38
Adaboost regressor	40.39	40.75	49.66	50.16	21.36	21.69	25.52	25.83
Multilayer perceptron	0.59	1.73	0.84	2.50	0.29	0.93	0.42	1.38
Proposed model	1.64	1.47	2.16	2.06	0.94	0.89	1.25	1.24

## Data Availability

Not applicable.
